# 7-Meth­oxy-3-(4-meth­oxy­phen­yl)chroman-4-one

**DOI:** 10.1107/S1600536811055048

**Published:** 2012-01-07

**Authors:** Zhi-Yun Peng, Xiao-Yang Liu, Ye-Ling Yang, Kai-Shuang Xiang, Zhu-Ping Xiao

**Affiliations:** a The Key Laboratory of Hunan Forest Products and Chemical Industry Engineering of Hunan Province, and College of Chemistry and Chemical Engineering, Jishou University, Jishou 416000, People’s Republic of China

## Abstract

The asymmetric unit of the title compound, C_17_H_16_O_4_, contains two crystallographically independent mol­ecules with different absolute configurations.

## Related literature

Flavonoids are thought to have protective effects against cardiovascular diseases, cancers and other age-related diseases due to their high anti­oxidant capacity, see: Zhang *et al.* (2008 ▶). For our efforts to synthesize derivatives of flavonoids for urease inhibitors and anti­bacterial activity screening, see: Xiao *et al.* (2010 ▶, 2011 ▶).
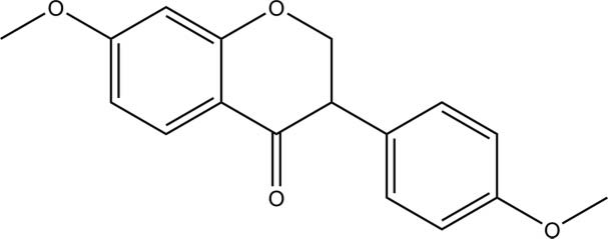


## Experimental

### 

#### Crystal data

C_17_H_16_O_4_
*M* *_r_* = 284.30
Orthorhombic, 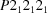

*a* = 10.601 (2) Å
*b* = 15.762 (4) Å
*c* = 16.793 (4) Å
*V* = 2805.9 (11) Å^3^
*Z* = 8
Mo *K*α radiation
μ = 0.10 mm^−1^
*T* = 296 K
0.20 × 0.20 × 0.10 mm

#### Data collection

Bruker SMART APEX CCD diffractometer
Absorption correction: multi-scan (*SADABS*; Sheldrick, 1996 ▶) *T*_min_ = 0.981, *T*_max_ = 0.991
14633 measured reflections
5752 independent reflections
3699 reflections with *I* > 2σ(*I*)
*R*_int_ = 0.034

#### Refinement

*R*[*F* ^2^ > 2σ(*F* ^2^)] = 0.053
*wR*(*F* ^2^) = 0.141
*S* = 1.03
5752 reflections
383 parameters
H-atom parameters constrained
Δρ_max_ = 0.41 e Å^−3^
Δρ_min_ = −0.16 e Å^−3^

### 

Data collection: *SMART* (Bruker, 2007 ▶); cell refinement: *SAINT* (Bruker, 2007 ▶); data reduction: *SAINT*; program(s) used to solve structure: *SHELXS97* (Sheldrick, 2008 ▶); program(s) used to refine structure: *SHELXL97* (Sheldrick, 2008 ▶); molecular graphics: *SHELXTL* (Sheldrick, 2008 ▶); software used to prepare material for publication: *SHELXTL*.

## Supplementary Material

Crystal structure: contains datablock(s) global, I. DOI: 10.1107/S1600536811055048/aa2036sup1.cif

Structure factors: contains datablock(s) I. DOI: 10.1107/S1600536811055048/aa2036Isup2.hkl

Supplementary material file. DOI: 10.1107/S1600536811055048/aa2036Isup3.cml

Additional supplementary materials:  crystallographic information; 3D view; checkCIF report

## Figures and Tables

**Table 1:** Hydrogen-bond geometry (Å, °)

*D*—H⋯*A*	*D*—H	H⋯*A*	*D*⋯*A*	*D*—H⋯*A*
C33—H33*B*⋯O6^i^	0.96	2.47	3.278 (4)	141

Symmetry code: (i) 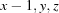.

**Fig. 1.**
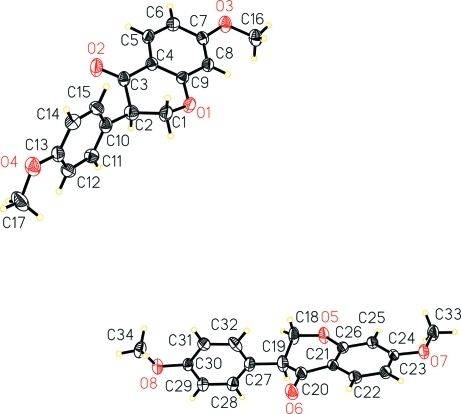
Molecular structure of the title compound. Thermal displacement ellipsoids are drawn at the 30% probability level.

**Fig. 2.**
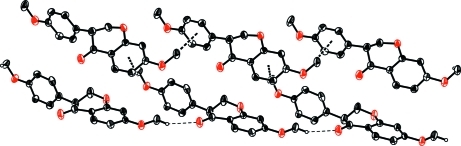
Packing diagram.

## supplementary crystallographic information

### Comment

Flavonoids are thought to have protective effects against cardiovascular diseases, cancers, and other age-related diseases due to their high antioxidant capacity both *in vivo* and *in vitro* systems (Zhang, *et al.*, 2008). Recently, we focused our efforts to synthesize derivatives of flavonoids for urease inhibitors and antibacterial activity screening (Xiao, *et al.*, 2010 and 2011).

The crystal structure of the title compound, 7-methoxy-3-(4-methoxyphenyl)chroman-4-one, contains two crystallographically independent molecules (Fig. 1) in the asymmetric unit. They are a pair of enantiomers, and we define C1 to C17 as molecule A, while C18 to C34 as molecule B. In molecule A, the ring C4/C5/C6/C7/C8/C9 makes a dihedral angle of 72.74 (9) ° with the ring C10/C11/C12/C13/C14/C15. However, in molecule B, the corresponding dihedral angle is 83.32 (11) °.

### Experimental

LiAlH_4_ (5.0 mmol) and AlCl_3_ (6 mmol) was dissolved in THF (10 ml), and 6 mmol of 7-methoxy-3-(4-methoxyphenyl)-4*H*-chromen-4-one was subsequently added. The mixture was stirred at 273 K for 3 h, and extracted with ethylacetate followed by addition of water (5 ml). After removal of the solvent, the resulting residue was purified over a silica gel column eluting with etrylacetate-petroleum ether (1:1). The crystals suitable for single-crystal structure determination of the title compound were grown from ethylacetate-petroleum ether at room temperature by slow evaporation.

### Refinement

All H atoms were placed in geometrically idealized positions and constrained to ride on their parent atoms with C—H = 0.93 Å for aromatic H atoms, 0.96 Å for CH_3_ type H atoms, 0.97 Å for CH_2_ type H atoms and 0.98 Å for CH type H atom, respectively. *U*_iso_(H) values were set at 1.5 times *U*_eq_(C) for methyl H atoms, and 1.2 times for the rest H atoms.

### Crystal data

**Table:**

C_17_H_16_O_4_	*F*(000) = 1200
*M**_r_* = 284.30	*D*_x_ = 1.346 Mg m^−^^3^
Orthorhombic, *P*2_1_2_1_2_1_	Mo *K*α radiation, λ = 0.71073 Å
Hall symbol: P 2ac 2ab	Cell parameters from 3901 reflections
*a* = 10.601 (2) Å	θ = 2.5–26.0°
*b* = 15.762 (4) Å	µ = 0.10 mm^−^^1^
*c* = 16.793 (4) Å	*T* = 296 K
*V* = 2805.9 (11) Å^3^	Block, colorless
*Z* = 8	0.20 × 0.20 × 0.10 mm

### Data collection

**Table:**

Bruker SMART APEX CCD diffractometer	5752 independent reflections
Radiation source: fine-focus sealed tube	3699 reflections with *I* > 2σ(*I*)
graphite	*R*_int_ = 0.034
φ and ω scan	θ_max_ = 26.5°, θ_min_ = 2.3°
Absorption correction: multi-scan (*SADABS*; Sheldrick, 1996)	*h* = −12→13
*T*_min_ = 0.981, *T*_max_ = 0.991	*k* = −19→19
14633 measured reflections	*l* = −14→21

### Refinement

**Table:**

Refinement on *F*^2^	Primary atom site location: structure-invariant direct methods
Least-squares matrix: full	Secondary atom site location: difference Fourier map
*R*[*F*^2^ > 2σ(*F*^2^)] = 0.053	Hydrogen site location: inferred from neighbouring sites
*wR*(*F*^2^) = 0.141	H-atom parameters constrained
*S* = 1.03	*w* = 1/[σ^2^(*F*_o_^2^) + (0.0682*P*)^2^ + 0.1397*P*] where *P* = (*F*_o_^2^ + 2*F*_c_^2^)/3
5752 reflections	(Δ/σ)_max_ < 0.001
383 parameters	Δρ_max_ = 0.41 e Å^−^^3^
0 restraints	Δρ_min_ = −0.16 e Å^−^^3^

### Special details

**Table:**

Geometry. All s.u.'s (except the s.u. in the dihedral angle between two l.s. planes) are estimated using the full covariance matrix. The cell s.u.'s are taken into account individually in the estimation of s.u.'s in distances, angles and torsion angles; correlations between s.u.'s in cell parameters are only used when they are defined by crystal symmetry. An approximate (isotropic) treatment of cell s.u.'s is used for estimating s.u.'s involving l.s. planes.
Refinement. Refinement of *F*^2^ against ALL reflections. The weighted *R*-factor *wR* and goodness of fit *S* are based on *F*^2^, conventional *R*-factors *R* are based on *F*, with *F* set to zero for negative *F*^2^. The threshold expression of *F*^2^ > σ(*F*^2^) is used only for calculating *R*-factors(gt) *etc*. and is not relevant to the choice of reflections for refinement. *R*-factors based on *F*^2^ are statistically about twice as large as those based on *F*, and *R*- factors based on ALL data will be even larger.

### Fractional atomic coordinates and isotropic or equivalent isotropic displacement parameters (Å^2^)

**Table:**

	*x*	*y*	*z*	*U*_iso_*/*U*_eq_	
C1	0.5166 (3)	0.7672 (2)	0.2776 (2)	0.0665 (10)	
H1A	0.5125	0.7484	0.2226	0.080*	
H1B	0.5336	0.7178	0.3103	0.080*	
C2	0.6228 (3)	0.8276 (2)	0.2860 (2)	0.0613 (9)	
H2	0.6243	0.8451	0.3420	0.074*	
C3	0.5957 (3)	0.90714 (18)	0.2376 (2)	0.0508 (8)	
C4	0.4627 (3)	0.93083 (18)	0.23531 (18)	0.0456 (7)	
C5	0.4228 (3)	1.00835 (18)	0.20398 (19)	0.0536 (8)	
H5	0.4821	1.0445	0.1812	0.064*	
C6	0.2990 (3)	1.03259 (19)	0.20583 (19)	0.0557 (8)	
H6	0.2750	1.0847	0.1848	0.067*	
C7	0.2092 (3)	0.97925 (19)	0.23918 (18)	0.0491 (7)	
C8	0.2442 (3)	0.90182 (19)	0.27018 (19)	0.0515 (8)	
H8	0.1842	0.8660	0.2925	0.062*	
C9	0.3703 (3)	0.87803 (18)	0.26759 (18)	0.0458 (7)	
C10	0.7500 (3)	0.78672 (18)	0.2689 (2)	0.0544 (8)	
C11	0.7875 (3)	0.75939 (19)	0.1948 (2)	0.0568 (8)	
H11	0.7347	0.7685	0.1513	0.068*	
C12	0.9017 (3)	0.71863 (19)	0.1831 (2)	0.0584 (8)	
H12	0.9246	0.6999	0.1326	0.070*	
C13	0.9818 (3)	0.70588 (19)	0.2476 (2)	0.0554 (9)	
C14	0.9440 (3)	0.73214 (19)	0.3223 (2)	0.0568 (8)	
H14	0.9961	0.7232	0.3661	0.068*	
C15	0.8298 (3)	0.7713 (2)	0.33210 (19)	0.0556 (8)	
H15	0.8053	0.7880	0.3829	0.067*	
C16	−0.0074 (3)	0.9543 (2)	0.2701 (2)	0.0706 (10)	
H16A	−0.0069	0.9000	0.2441	0.106*	
H16B	−0.0882	0.9807	0.2628	0.106*	
H16C	0.0084	0.9468	0.3259	0.106*	
C17	1.1718 (3)	0.6448 (3)	0.2977 (3)	0.0954 (14)	
H17A	1.1985	0.6952	0.3249	0.143*	
H17B	1.2444	0.6142	0.2792	0.143*	
H17C	1.1243	0.6097	0.3335	0.143*	
C18	0.1377 (3)	0.5956 (2)	1.0117 (3)	0.0792 (12)	
H18A	0.1493	0.6508	1.0362	0.095*	
H18B	0.1475	0.6027	0.9547	0.095*	
C19	0.2374 (3)	0.5387 (2)	1.0404 (3)	0.0736 (11)	
H19	0.2248	0.5342	1.0980	0.088*	
C20	0.2169 (3)	0.45049 (19)	1.0078 (2)	0.0563 (8)	
C21	0.0856 (3)	0.42614 (17)	0.99789 (17)	0.0421 (7)	
C22	0.0498 (3)	0.34237 (18)	0.98230 (19)	0.0518 (8)	
H22	0.1118	0.3023	0.9711	0.062*	
C23	−0.0736 (3)	0.31797 (17)	0.9831 (2)	0.0525 (8)	
H23	−0.0951	0.2618	0.9730	0.063*	
C24	−0.1670 (3)	0.37726 (17)	0.99896 (17)	0.0440 (7)	
C25	−0.1365 (3)	0.46103 (17)	1.01252 (18)	0.0461 (7)	
H25	−0.1994	0.5010	1.0218	0.055*	
C26	−0.0111 (3)	0.48479 (16)	1.01209 (17)	0.0450 (7)	
C27	0.3690 (3)	0.5740 (2)	1.0297 (2)	0.0600 (9)	
C28	0.4459 (3)	0.58703 (19)	1.0935 (2)	0.0600 (9)	
H28	0.4169	0.5732	1.1442	0.072*	
C29	0.5642 (3)	0.6198 (2)	1.0852 (2)	0.0539 (8)	
H29	0.6145	0.6277	1.1299	0.065*	
C30	0.6100 (3)	0.64138 (16)	1.01120 (18)	0.0449 (7)	
C31	0.5366 (3)	0.6276 (2)	0.9448 (2)	0.0580 (8)	
H31	0.5664	0.6409	0.8942	0.070*	
C32	0.4158 (3)	0.5929 (2)	0.9551 (2)	0.0674 (10)	
H32	0.3661	0.5824	0.9106	0.081*	
C33	−0.3882 (3)	0.4047 (2)	1.0076 (3)	0.0741 (11)	
H33A	−0.3813	0.4334	1.0579	0.111*	
H33B	−0.4667	0.3744	1.0054	0.111*	
H33C	−0.3854	0.4456	0.9652	0.111*	
C34	0.7894 (3)	0.6869 (2)	0.9356 (2)	0.0702 (10)	
H34A	0.7964	0.6330	0.9092	0.105*	
H34B	0.8720	0.7102	0.9438	0.105*	
H34C	0.7407	0.7249	0.9032	0.105*	
O1	0.39795 (19)	0.80063 (12)	0.29927 (15)	0.0646 (7)	
O2	0.6782 (2)	0.95173 (15)	0.21016 (18)	0.0815 (8)	
O3	0.0888 (2)	1.00702 (14)	0.23637 (15)	0.0665 (6)	
O4	1.0946 (2)	0.66753 (16)	0.23146 (17)	0.0768 (7)	
O5	0.01306 (19)	0.56791 (13)	1.02714 (16)	0.0678 (7)	
O6	0.3028 (2)	0.39876 (14)	1.00363 (17)	0.0798 (8)	
O7	−0.28651 (18)	0.34673 (12)	0.99912 (14)	0.0607 (6)	
O8	0.72870 (19)	0.67584 (14)	1.01015 (13)	0.0604 (6)	

### Atomic displacement parameters (Å^2^)

**Table:**

	*U*^11^	*U*^22^	*U*^33^	*U*^12^	*U*^13^	*U*^23^
C1	0.049 (2)	0.0531 (18)	0.098 (3)	0.0031 (16)	0.010 (2)	0.0168 (18)
C2	0.053 (2)	0.0519 (17)	0.079 (2)	0.0071 (16)	−0.0011 (18)	0.0017 (17)
C3	0.0434 (19)	0.0398 (15)	0.069 (2)	−0.0033 (14)	0.0028 (16)	−0.0044 (14)
C4	0.0432 (19)	0.0422 (15)	0.0514 (18)	−0.0008 (13)	−0.0010 (14)	−0.0022 (13)
C5	0.0491 (19)	0.0462 (16)	0.065 (2)	−0.0066 (15)	0.0050 (16)	0.0071 (15)
C6	0.054 (2)	0.0427 (16)	0.071 (2)	0.0057 (15)	0.0004 (17)	0.0035 (15)
C7	0.0407 (18)	0.0502 (18)	0.0564 (18)	0.0023 (14)	−0.0006 (15)	−0.0048 (14)
C8	0.0416 (18)	0.0538 (18)	0.0592 (19)	−0.0020 (14)	0.0056 (15)	0.0022 (15)
C9	0.0477 (19)	0.0412 (15)	0.0484 (17)	−0.0004 (13)	0.0015 (14)	0.0009 (13)
C10	0.0461 (19)	0.0424 (16)	0.075 (2)	−0.0022 (14)	0.0049 (17)	0.0001 (16)
C11	0.054 (2)	0.0546 (18)	0.062 (2)	−0.0024 (16)	−0.0137 (18)	0.0050 (16)
C12	0.067 (2)	0.0531 (17)	0.055 (2)	−0.0020 (17)	0.0034 (18)	−0.0044 (15)
C13	0.0430 (19)	0.0464 (17)	0.077 (3)	−0.0015 (15)	0.0007 (17)	0.0008 (16)
C14	0.055 (2)	0.0577 (18)	0.058 (2)	0.0026 (16)	−0.0096 (17)	0.0028 (16)
C15	0.057 (2)	0.0580 (18)	0.0517 (19)	0.0033 (16)	0.0005 (17)	−0.0013 (15)
C16	0.0416 (19)	0.075 (2)	0.095 (3)	0.0097 (18)	0.010 (2)	−0.004 (2)
C17	0.055 (2)	0.081 (3)	0.150 (4)	0.017 (2)	−0.024 (3)	0.009 (3)
C18	0.044 (2)	0.0441 (17)	0.149 (4)	−0.0088 (15)	0.001 (2)	−0.013 (2)
C19	0.046 (2)	0.057 (2)	0.118 (3)	−0.0070 (17)	0.000 (2)	−0.009 (2)
C20	0.0426 (19)	0.0464 (16)	0.080 (2)	0.0013 (15)	−0.0040 (18)	0.0017 (16)
C21	0.0363 (16)	0.0410 (14)	0.0489 (17)	0.0024 (12)	−0.0016 (14)	0.0010 (12)
C22	0.0475 (19)	0.0409 (16)	0.067 (2)	0.0088 (14)	−0.0038 (16)	−0.0035 (14)
C23	0.0496 (19)	0.0340 (14)	0.074 (2)	−0.0020 (13)	−0.0088 (17)	−0.0042 (14)
C24	0.0354 (16)	0.0421 (14)	0.0545 (18)	−0.0003 (12)	−0.0093 (14)	0.0032 (14)
C25	0.0409 (17)	0.0384 (14)	0.0590 (19)	0.0038 (12)	−0.0020 (14)	−0.0049 (13)
C26	0.0454 (18)	0.0374 (14)	0.0521 (18)	0.0012 (13)	−0.0018 (14)	−0.0023 (13)
C27	0.045 (2)	0.0467 (18)	0.088 (3)	−0.0048 (15)	0.0068 (19)	−0.0004 (18)
C28	0.056 (2)	0.055 (2)	0.069 (2)	−0.0074 (17)	0.0047 (18)	−0.0027 (16)
C29	0.0466 (19)	0.0557 (18)	0.059 (2)	−0.0058 (15)	0.0018 (16)	−0.0064 (15)
C30	0.0397 (17)	0.0380 (14)	0.057 (2)	−0.0044 (12)	0.0017 (15)	−0.0064 (14)
C31	0.055 (2)	0.064 (2)	0.055 (2)	−0.0058 (17)	−0.0069 (17)	0.0038 (16)
C32	0.049 (2)	0.078 (2)	0.076 (3)	0.0067 (19)	−0.023 (2)	−0.014 (2)
C33	0.0402 (19)	0.061 (2)	0.121 (3)	−0.0022 (16)	0.005 (2)	0.002 (2)
C34	0.060 (2)	0.069 (2)	0.082 (3)	−0.0110 (19)	0.023 (2)	−0.0022 (18)
O1	0.0452 (13)	0.0520 (12)	0.0967 (18)	0.0035 (10)	0.0122 (12)	0.0219 (12)
O2	0.0483 (14)	0.0606 (14)	0.136 (2)	−0.0077 (12)	0.0038 (15)	0.0222 (15)
O3	0.0434 (13)	0.0552 (12)	0.1010 (18)	0.0060 (11)	0.0061 (13)	0.0001 (12)
O4	0.0524 (14)	0.0700 (15)	0.108 (2)	0.0136 (13)	0.0108 (15)	0.0035 (14)
O5	0.0392 (13)	0.0409 (11)	0.123 (2)	−0.0001 (9)	0.0027 (13)	−0.0177 (12)
O6	0.0435 (14)	0.0604 (14)	0.135 (2)	0.0086 (11)	−0.0034 (16)	0.0031 (15)
O7	0.0391 (12)	0.0472 (11)	0.0958 (17)	−0.0052 (10)	−0.0051 (12)	−0.0040 (11)
O8	0.0471 (13)	0.0733 (13)	0.0607 (14)	−0.0151 (11)	0.0063 (11)	−0.0113 (11)

### Geometric parameters (Å, °)

**Table:**

C1—O1	1.411 (4)	C18—O5	1.415 (4)
C1—C2	1.482 (4)	C18—C19	1.467 (5)
C1—H1A	0.9700	C18—H18A	0.9700
C1—H1B	0.9700	C18—H18B	0.9700
C2—C3	1.521 (5)	C19—C20	1.511 (5)
C2—C10	1.523 (4)	C19—C27	1.512 (5)
C2—H2	0.9800	C19—H19	0.9800
C3—O2	1.213 (4)	C20—O6	1.224 (3)
C3—C4	1.459 (4)	C20—C21	1.453 (4)
C4—C9	1.395 (4)	C21—C22	1.399 (4)
C4—C5	1.396 (4)	C21—C26	1.401 (4)
C5—C6	1.368 (4)	C22—C23	1.364 (4)
C5—H5	0.9300	C22—H22	0.9300
C6—C7	1.388 (4)	C23—C24	1.388 (4)
C6—H6	0.9300	C23—H23	0.9300
C7—O3	1.350 (3)	C24—O7	1.355 (3)
C7—C8	1.378 (4)	C24—C25	1.378 (4)
C8—C9	1.389 (4)	C25—C26	1.381 (4)
C8—H8	0.9300	C25—H25	0.9300
C9—O1	1.363 (3)	C26—O5	1.359 (3)
C10—C11	1.376 (5)	C27—C28	1.362 (5)
C10—C15	1.379 (4)	C27—C32	1.380 (5)
C11—C12	1.384 (4)	C28—C29	1.364 (4)
C11—H11	0.9300	C28—H28	0.9300
C12—C13	1.391 (5)	C29—C30	1.376 (4)
C12—H12	0.9300	C29—H29	0.9300
C13—O4	1.366 (4)	C30—O8	1.370 (3)
C13—C14	1.381 (5)	C30—C31	1.377 (4)
C14—C15	1.368 (4)	C31—C32	1.403 (4)
C14—H14	0.9300	C31—H31	0.9300
C15—H15	0.9300	C32—H32	0.9300
C16—O3	1.432 (4)	C33—O7	1.421 (4)
C16—H16A	0.9600	C33—H33A	0.9600
C16—H16B	0.9600	C33—H33B	0.9600
C16—H16C	0.9600	C33—H33C	0.9600
C17—O4	1.427 (5)	C34—O8	1.419 (4)
C17—H17A	0.9600	C34—H34A	0.9600
C17—H17B	0.9600	C34—H34B	0.9600
C17—H17C	0.9600	C34—H34C	0.9600
			
O1—C1—C2	114.3 (3)	O5—C18—H18B	108.5
O1—C1—H1A	108.7	C19—C18—H18B	108.5
C2—C1—H1A	108.7	H18A—C18—H18B	107.5
O1—C1—H1B	108.7	C18—C19—C20	109.8 (3)
C2—C1—H1B	108.7	C18—C19—C27	113.7 (3)
H1A—C1—H1B	107.6	C20—C19—C27	115.4 (3)
C1—C2—C3	109.6 (3)	C18—C19—H19	105.7
C1—C2—C10	112.5 (3)	C20—C19—H19	105.7
C3—C2—C10	114.5 (3)	C27—C19—H19	105.7
C1—C2—H2	106.6	O6—C20—C21	122.0 (3)
C3—C2—H2	106.6	O6—C20—C19	121.8 (3)
C10—C2—H2	106.6	C21—C20—C19	115.0 (3)
O2—C3—C4	122.6 (3)	C22—C21—C26	117.2 (3)
O2—C3—C2	123.0 (3)	C22—C21—C20	122.0 (3)
C4—C3—C2	114.0 (3)	C26—C21—C20	120.5 (2)
C9—C4—C5	117.1 (3)	C23—C22—C21	121.6 (3)
C9—C4—C3	121.0 (3)	C23—C22—H22	119.2
C5—C4—C3	121.8 (3)	C21—C22—H22	119.2
C6—C5—C4	121.8 (3)	C22—C23—C24	119.8 (3)
C6—C5—H5	119.1	C22—C23—H23	120.1
C4—C5—H5	119.1	C24—C23—H23	120.1
C5—C6—C7	119.9 (3)	O7—C24—C25	124.0 (3)
C5—C6—H6	120.1	O7—C24—C23	115.4 (2)
C7—C6—H6	120.1	C25—C24—C23	120.6 (3)
O3—C7—C8	123.7 (3)	C24—C25—C26	119.0 (3)
O3—C7—C6	116.0 (3)	C24—C25—H25	120.5
C8—C7—C6	120.3 (3)	C26—C25—H25	120.5
C7—C8—C9	119.1 (3)	O5—C26—C25	116.2 (2)
C7—C8—H8	120.4	O5—C26—C21	122.0 (3)
C9—C8—H8	120.4	C25—C26—C21	121.8 (2)
O1—C9—C8	115.9 (3)	C28—C27—C32	117.8 (3)
O1—C9—C4	122.4 (3)	C28—C27—C19	121.0 (3)
C8—C9—C4	121.8 (3)	C32—C27—C19	121.2 (4)
C11—C10—C15	117.7 (3)	C27—C28—C29	121.8 (3)
C11—C10—C2	124.0 (3)	C27—C28—H28	119.1
C15—C10—C2	118.3 (3)	C29—C28—H28	119.1
C10—C11—C12	121.7 (3)	C28—C29—C30	120.8 (3)
C10—C11—H11	119.2	C28—C29—H29	119.6
C12—C11—H11	119.2	C30—C29—H29	119.6
C11—C12—C13	119.4 (3)	O8—C30—C29	115.7 (3)
C11—C12—H12	120.3	O8—C30—C31	124.8 (3)
C13—C12—H12	120.3	C29—C30—C31	119.5 (3)
O4—C13—C14	124.5 (3)	C30—C31—C32	118.5 (3)
O4—C13—C12	116.3 (3)	C30—C31—H31	120.8
C14—C13—C12	119.1 (3)	C32—C31—H31	120.8
C15—C14—C13	120.1 (3)	C27—C32—C31	121.7 (3)
C15—C14—H14	120.0	C27—C32—H32	119.2
C13—C14—H14	120.0	C31—C32—H32	119.2
C14—C15—C10	122.0 (3)	O7—C33—H33A	109.5
C14—C15—H15	119.0	O7—C33—H33B	109.5
C10—C15—H15	119.0	H33A—C33—H33B	109.5
O3—C16—H16A	109.5	O7—C33—H33C	109.5
O3—C16—H16B	109.5	H33A—C33—H33C	109.5
H16A—C16—H16B	109.5	H33B—C33—H33C	109.5
O3—C16—H16C	109.5	O8—C34—H34A	109.5
H16A—C16—H16C	109.5	O8—C34—H34B	109.5
H16B—C16—H16C	109.5	H34A—C34—H34B	109.5
O4—C17—H17A	109.5	O8—C34—H34C	109.5
O4—C17—H17B	109.5	H34A—C34—H34C	109.5
H17A—C17—H17B	109.5	H34B—C34—H34C	109.5
O4—C17—H17C	109.5	C9—O1—C1	115.2 (2)
H17A—C17—H17C	109.5	C7—O3—C16	118.1 (2)
H17B—C17—H17C	109.5	C13—O4—C17	117.3 (3)
O5—C18—C19	115.1 (3)	C26—O5—C18	116.1 (2)
O5—C18—H18A	108.5	C24—O7—C33	118.8 (2)
C19—C18—H18A	108.5	C30—O8—C34	118.4 (2)
			
O1—C1—C2—C3	−56.3 (4)	C19—C20—C21—C22	−168.2 (3)
O1—C1—C2—C10	175.0 (3)	O6—C20—C21—C26	172.5 (3)
C1—C2—C3—O2	−152.6 (3)	C19—C20—C21—C26	5.0 (5)
C10—C2—C3—O2	−25.1 (5)	C26—C21—C22—C23	−1.8 (5)
C1—C2—C3—C4	34.6 (4)	C20—C21—C22—C23	171.6 (3)
C10—C2—C3—C4	162.1 (3)	C21—C22—C23—C24	0.6 (5)
O2—C3—C4—C9	−179.9 (3)	C22—C23—C24—O7	−179.4 (3)
C2—C3—C4—C9	−7.0 (4)	C22—C23—C24—C25	1.2 (5)
O2—C3—C4—C5	−2.5 (5)	O7—C24—C25—C26	179.1 (3)
C2—C3—C4—C5	170.3 (3)	C23—C24—C25—C26	−1.6 (5)
C9—C4—C5—C6	1.1 (4)	C24—C25—C26—O5	−178.9 (3)
C3—C4—C5—C6	−176.3 (3)	C24—C25—C26—C21	0.3 (5)
C4—C5—C6—C7	−0.3 (5)	C22—C21—C26—O5	−179.4 (3)
C5—C6—C7—O3	−178.1 (3)	C20—C21—C26—O5	7.1 (5)
C5—C6—C7—C8	−0.2 (5)	C22—C21—C26—C25	1.4 (4)
O3—C7—C8—C9	177.7 (3)	C20—C21—C26—C25	−172.1 (3)
C6—C7—C8—C9	0.0 (5)	C18—C19—C27—C28	119.8 (4)
C7—C8—C9—O1	−179.9 (3)	C20—C19—C27—C28	−112.0 (4)
C7—C8—C9—C4	0.8 (5)	C18—C19—C27—C32	−61.1 (5)
C5—C4—C9—O1	179.5 (3)	C20—C19—C27—C32	67.1 (5)
C3—C4—C9—O1	−3.1 (5)	C32—C27—C28—C29	1.8 (5)
C5—C4—C9—C8	−1.3 (4)	C19—C27—C28—C29	−179.1 (3)
C3—C4—C9—C8	176.1 (3)	C27—C28—C29—C30	0.3 (5)
C1—C2—C10—C11	68.4 (4)	C28—C29—C30—O8	178.3 (3)
C3—C2—C10—C11	−57.7 (4)	C28—C29—C30—C31	−1.7 (4)
C1—C2—C10—C15	−107.5 (4)	O8—C30—C31—C32	−179.0 (3)
C3—C2—C10—C15	126.4 (3)	C29—C30—C31—C32	1.0 (4)
C15—C10—C11—C12	−0.7 (4)	C28—C27—C32—C31	−2.5 (5)
C2—C10—C11—C12	−176.6 (3)	C19—C27—C32—C31	178.4 (3)
C10—C11—C12—C13	−0.9 (4)	C30—C31—C32—C27	1.1 (5)
C11—C12—C13—O4	−178.1 (3)	C8—C9—O1—C1	163.5 (3)
C11—C12—C13—C14	1.8 (4)	C4—C9—O1—C1	−17.2 (4)
O4—C13—C14—C15	178.9 (3)	C2—C1—O1—C9	48.1 (4)
C12—C13—C14—C15	−1.0 (5)	C8—C7—O3—C16	2.3 (4)
C13—C14—C15—C10	−0.7 (5)	C6—C7—O3—C16	−179.9 (3)
C11—C10—C15—C14	1.6 (5)	C14—C13—O4—C17	7.3 (5)
C2—C10—C15—C14	177.7 (3)	C12—C13—O4—C17	−172.8 (3)
O5—C18—C19—C20	54.4 (5)	C25—C26—O5—C18	−168.2 (3)
O5—C18—C19—C27	−174.6 (3)	C21—C26—O5—C18	12.5 (4)
C18—C19—C20—O6	158.8 (4)	C19—C18—O5—C26	−44.7 (5)
C27—C19—C20—O6	28.7 (5)	C25—C24—O7—C33	5.1 (5)
C18—C19—C20—C21	−33.6 (4)	C23—C24—O7—C33	−174.3 (3)
C27—C19—C20—C21	−163.7 (3)	C29—C30—O8—C34	170.1 (3)
O6—C20—C21—C22	−0.7 (5)	C31—C30—O8—C34	−9.9 (4)

### Hydrogen-bond geometry (Å, °)

**Table:**

*D*—H···*A*	*D*—H	H···*A*	*D*···*A*	*D*—H···*A*
C33—H33B···O6^i^	0.96	2.47	3.278 (4)	141

Symmetry codes: (i) *x*−1, *y*, *z*.
